# A molecular perspective on starch metabolism in woody tissues

**DOI:** 10.1007/s00425-018-2954-2

**Published:** 2018-07-18

**Authors:** Henrique Noronha, Angélica Silva, Zhanwu Dai, Philippe Gallusci, Adamo D. Rombolà, Serge Delrot, Hernâni Gerós

**Affiliations:** 1 Centre of Molecular and Environmental Biology (CBMA), University of Minho, Braga, Portugal; 2 UMR EGFV, Bordeaux Science Agro, INRA, Université de Bordeaux, Villenave D’Ornon, France; 3 Department of Agricultural Sciences, Alma Mater Studiorum, University of Bologna, Bologna, Italy; 4 Centro de Investigação e de Tecnologias Agro-ambientais e Biológicas (CITAB), Vila Real, Portugal; 5 Centre of Biological Engineering (CEB), University of Minho, Braga, Portugal

**Keywords:** Starch metabolism, Amyloplast, Sugar transporter, Woody tissues

## Abstract

The elucidation of the molecular mechanisms of starch synthesis and mobilization in perennial woody tissues is of the utmost scientific and agricultural importance.

Starch is the main carbohydrate reserve in plants and is fundamental in human nutrition and several industrial processes. In leaves, starch accumulated during the day is degraded throughout the night and the resulting sugars, glucose and maltose, are exported to the cytosol by the specialized transmembrane translocators pGT and MEX, respectively. Nevertheless, the degradation of the starch granule is a complex process not completely elucidated. While the mechanisms of starch mobilization during germination in the dead endosperm of cereal seeds are well described, the molecular and biochemical mechanisms involved in starch storage in the heterotrophic tissues of woody plants and its utilization in spring and winter are still puzzling. It is known that some biochemical steps of starch synthesis are conserved in heterotrophic tissues and in the leaves, but some aspects are particular to sink organs. From an agronomic standpoint, the knowledge on starch storage and mobilization in woody tissues is pivotal to understand (and to optimize) some common practices in the field that modify source–sink relationships, such as pruning and defoliation. Soluble sugars resulting from starch are also pivotal to cold adaptation, and in several fruits, such as banana and kiwifruit, starch may provide soluble sugars during ripening. In this review, we explore the recent advances on the molecular mechanisms and regulations involved in starch synthesis and mobilization, with a focus on perennial woody tissues.

## Introduction

The transition from hunter-gathering to sedentary agriculture, also called the “Neolithic revolution”, occurred in different locations around the world. This revolution, that preceded the development of large and complex civilizations, was characterized by the cultivation of starchy staples, such as cereals (Hillman et al. 2001; Gepts 2004; Tanno and Willcox 2006). Nowadays, starch-accumulating crops, besides remaining a major food source for humans, are widely used as animal feedstock and in several industrial processes, including paper, textile and pharmaceutical industries (Zeeman et al. 2010). Also, starch is the main source of carbon accumulation in fruits such as banana (Zhang et al. 2005) and kiwifruit (Nardozza et al. 2013), but is also present in considerable amounts during the development and ripening of fruits that mainly store soluble sugars, such as tomato (Bias et al. 2014), apple (Li et al. 2012), pear (Mesa et al. 2016) and strawberry (Moing et al. 2001). Starch in crops such as maize has also been used to produce bioethanol in an effort to increase the bio-sustainability of fuel production (Smith 2008).

**Fig. 1:**
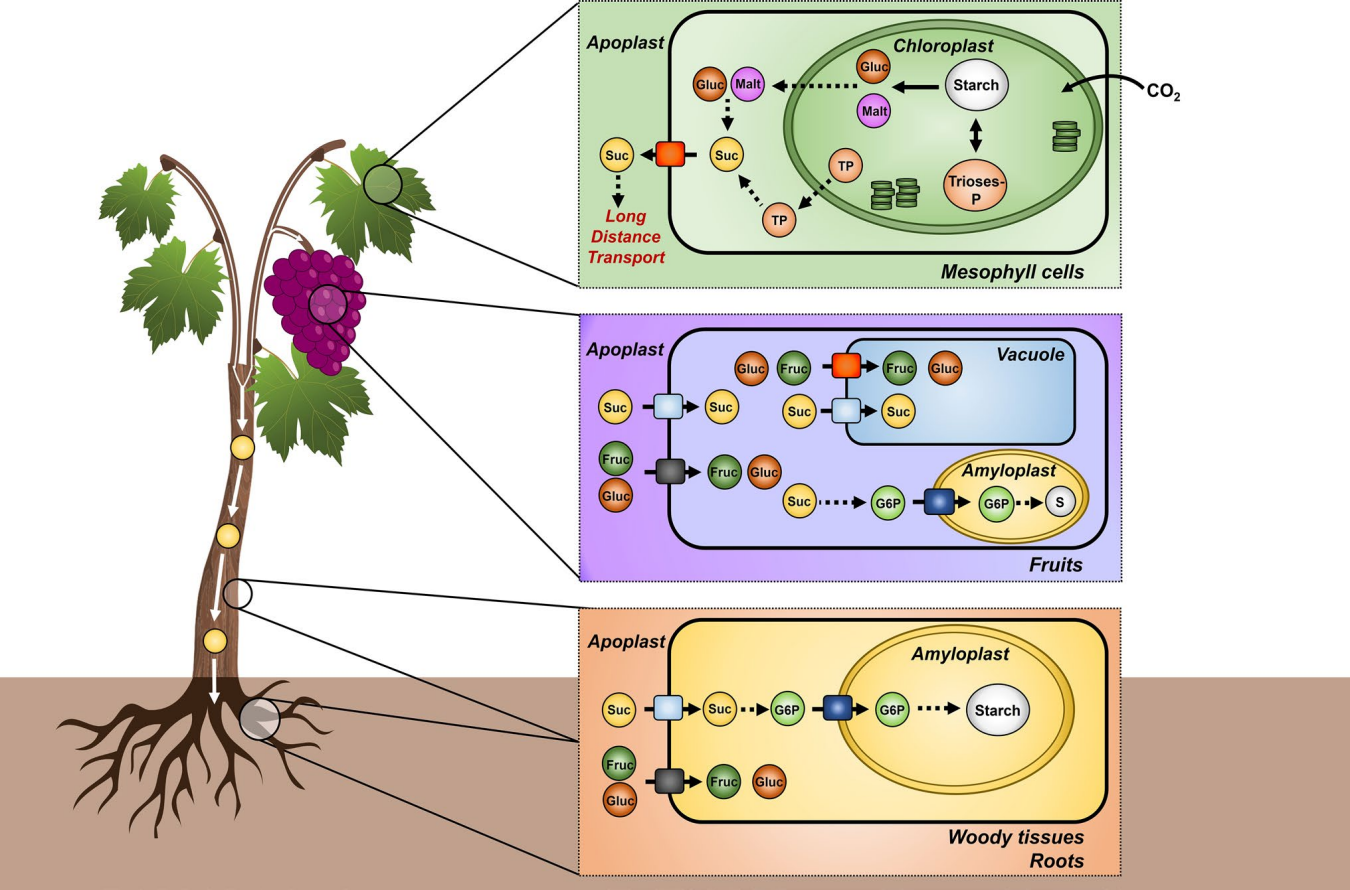
Long-distance transport of photoassimilates and starch synthesis in woody plants. Starch is synthesized in leaves inside chloroplasts from photosynthetic sugars. Following long-distance phloem transport, carbohydrates are accumulated as starch in the amyloplast of woody tissues and roots and as mono- or disaccharides in the vacuole Adapted from Lemoine et al. (2013)

Starch is synthesized in both autotrophic and heterotrophic tissues. In leaves, starch is synthesized in the chloroplast from the sugars produced by photosynthesis, and in sink tissues, including fruits, woody tissues and roots, starch is synthesized in the amyloplast after long-distance sugar transport via the phloem (Fig. 1). Several membrane proteins play pivotal roles in phloem loading and unloading with a major impact in plant development and productivity (Davies et al. 2012).

Starch is composed of two polymers of glucose, amylopectin and amylose, with the same type of glucosidic linkages that differ in their length and degree of branching. Amylopectin consists of chains of α-1,4-linked glucose units, branched by α-1,6-linkages (Smith et al. 1997), and has evolved from the ancestral capacity to make glycogen, the storage polysaccharide of animals, fungi, and bacteria that is a polymer much more branched than amylopectin (Copeland et al. 2009; Zeeman et al. 2010). Amylose is smaller than amylopectin and a linear polymer with α -1,4-linked glucose units (Slattery et al. 2000). The granule is organized in a layered structure of concentric lamellae, generally called growth rings that correspond to periodic depositions of starch (Smith 2001). In the granule matrix, amylopectin forms the amorphous and crystalline lamellae and amylose is thought to be mainly present in the amorphous lamellae (Buléon et al. 1998).

While the biochemical pathways involved in the synthesis of transient starch in the chloroplast are relatively well known, some aspects of its metabolism still remain elusive. For instance, the biochemical mechanisms involved in starch accumulation in the heterotrophic tissues of woody plants and its subsequent utilization in spring, and in winter in response to low-temperature, are far from being understood. Furthermore, some of this knowledge is pivotal to understand and optimize common agricultural practices that modify source–sink relationships and impact the amount of carbohydrate reserves in heterotrophic tissues.

In this review, the topic of starch synthesis and mobilization in auto- and heterotrophic tissues is approached from a molecular perspective, paying special attention to current knowledge in perennial woody tissues.

## Starch synthesis and mobilization in leaves

**Fig. 2:**
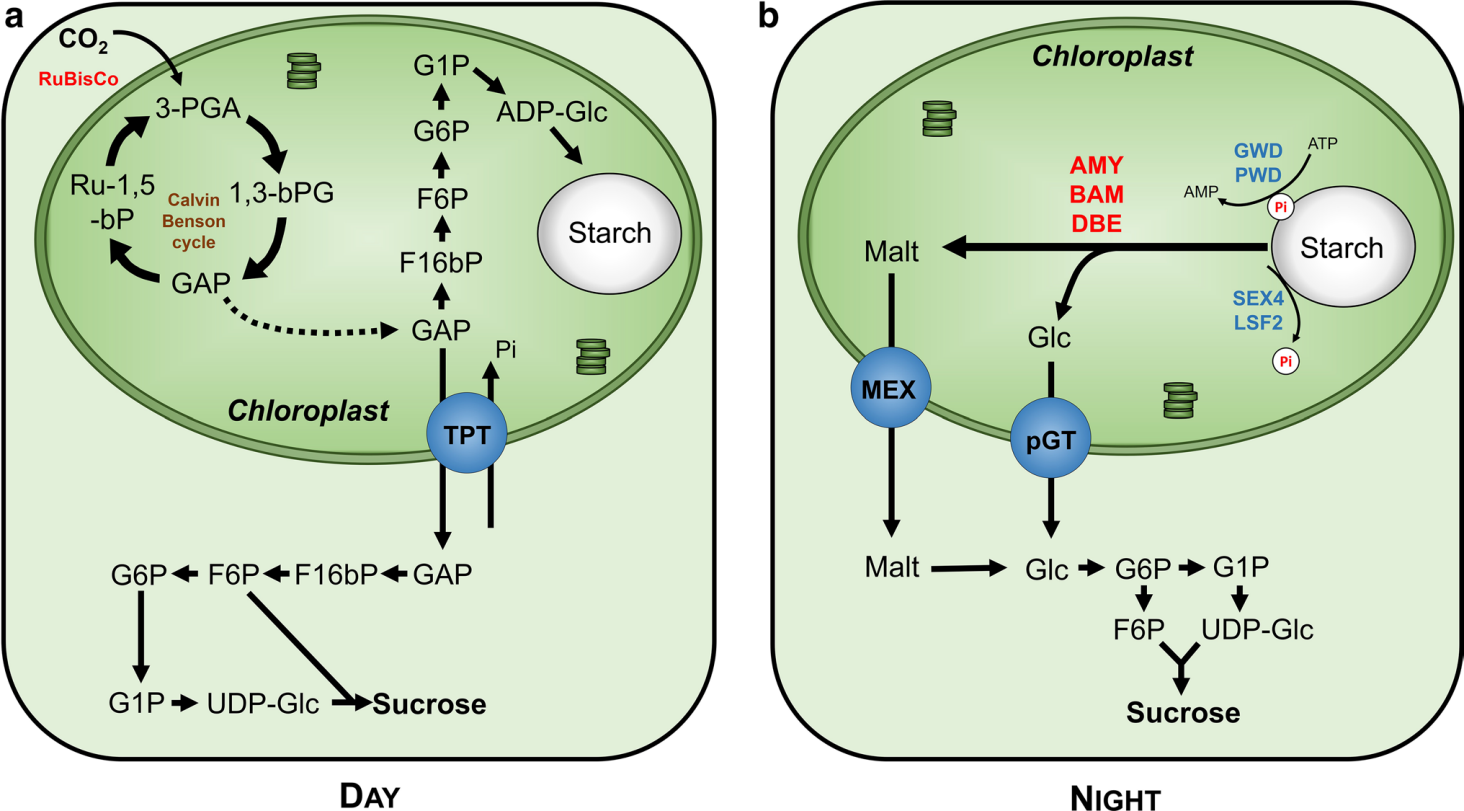
Starch synthesis and mobilization in autotrophic tissues. **a** During the day, carbon from atmospheric CO_2_ is assimilated in the Calvin–Benson cycle producing triose-phosphate, which are used to synthesize starch in the chloroplast and sucrose in the cytosol, after translocation through TPT. **b** During the night, starch is degraded by the coordinated action of α- and β-amylases, and the debranching enzymes (isoamylase and limit dextrinase), and the resulting glucose and maltose are exported to the cytosol by the MEX and pGT. In the cytosol, sucrose is synthesized to sustain heterotrophic tissues. *F16bP* fructose 1,6-bisphosphate, *F6P* fructose-6-phosphate, *G1P* glucose-1-phosphate, *G6P* glucose-6-phosphate, *Gluc* glucose, *MEX* maltose transporter, *pGT* plastidic glucose transporter, *Pi* inorganic phosphate, *TPT* triose-phosphate/phosphate translocator, *UDP-Glc* uridine diphosphate-glucose, *ADP-Glc* adenosine diphosphate-glucose, *GAP* Glyceraldehyde-3-phosphate, *ATP* Adenosine triphosphate, *3-PGA* 3-phosphoglyceric acid, *1,3-bPG* 1,3-bisphosphoglyceric acid, *Ru-1,5-bP* ribulose 1,5-bisphosphate

During the day, the Calvin–Benson cycle depends on the products of the photosynthetic light reactions, ATP and NADPH, to fix atmospheric CO_2_ into the form of triose-phosphate (triose-P; for a review see Raines (2003) and Bauwe et al. (2010)). These intermediates are mainly used for transient starch synthesis but are also translocated to the cytosol, via a triose-phosphate/phosphate translocator (TPT), and used as precursors for sucrose formation to fuel the metabolism during the day (Fig. 2). Inside the chloroplast, triose-P are converted into ADP-glucose and added to the starch granule by the coordinated action of several starch synthases (SS).

The degradation of the starch granule is a complex process that still is not completely elucidated (for a review see Zeeman et al. 2010; Andriotis et al. 2016a). In Arabidopsis, approximately 50% of carbon assimilated during the day is stored in the chloroplast in the form of starch, which is degraded during the night to feed the metabolism (Fig. 2; Zeeman and Rees 1999; Zeeman et al. 2007). β-amylases (BAM) hydrolyze the linear chains and release maltose from exposed non-reducing ends but are unable to hydrolyze amylopectin branching points, which are cleaved by debranching enzymes (DBE) (Zeeman et al. 2010). The role of BAM in starch degradation was elucidated in *starch excess* (*sex*) potato mutant where a plastidic BAM is downregulated (Scheidig et al. 2002). Furthermore, in *Arabidopsis thaliana*, DBEs, isoamylase (ISA) and limit dextrinase (LDA), are also involved in starch degradation because *isa3* and *isa3/lda* knock-out mutants display *sex* phenotypes. The role of α-amylases (AMY) in starch degradation in the leaves is still unclear and seems to vary between species. For instance, the Arabidopsis mutant *amy3* does not display any *sex* phenotype (Yu et al. 2005) but rice *α*-*amylase I*-*1* knock-out plants accumulate low levels of starch in the leaves (Asatsuma et al. 2005).

One important factor regulating starch degradation is the reversible phosphorylation of the glucans at the surface of the granule (Zeeman et al. 2010; MacNeill et al. 2017). The action of glucan, water dikinase (GWD) and phosphoglucan, water dikinase (PWD), which add phosphate groups from ATP to the glucosyl residues, and of phosphoglucan phosphatases, which remove the phosphate groups, allow the solubilization of the granule surface, facilitating the activity of the amylolytic enzymes. These enzymes are fundamental for starch degradation as shown in the Arabidopsis mutants *sex1* (loss of GWD; Yu et al. 2001) and *pwd* (loss of PWD; Baunsgaard et al. 2005). Also, it has been shown that the removal of the phosphate groups, by SEX4 and LSF1 (like SEX FOUR), is necessary for complete starch degradation (Zeeman et al. 2010). For instance, the loss of *AtSEX4* phosphatase reduces starch degradation in Arabidopsis leaves (Niittylä et al. 2006) and *lsf1* mutants show a starch excess phenotype and reduced rates of starch degradation (Comparot-Moss et al. 2010).

The final products of starch degradation in the chloroplasts during the night are mainly maltose with some glucose (Weise et al. 2004) that must be exported to the cytosol to allow a continuous supply of carbon (Fig. 2). Maltose is exported to the cytosol by the plastidic maltose transporter (MEX; Niittylä et al. 2004), which was identified following the characterization of *Arabidopsis thaliana maltose excess* mutant that displays an increased accumulation of maltose in leaves during the night. Furthermore, evidence of glucose transport across the chloroplast envelope was obtained by Schäfer et al. (1977) several years before the identification of the plastidic glucose translocator (pGT) (Weber et al. 2000). In the cytosol, maltose and glucose are converted to sucrose, the most common carbohydrate for long-distance sugar transport.

## Starch synthesis in sink tissues

**Fig. 3:**
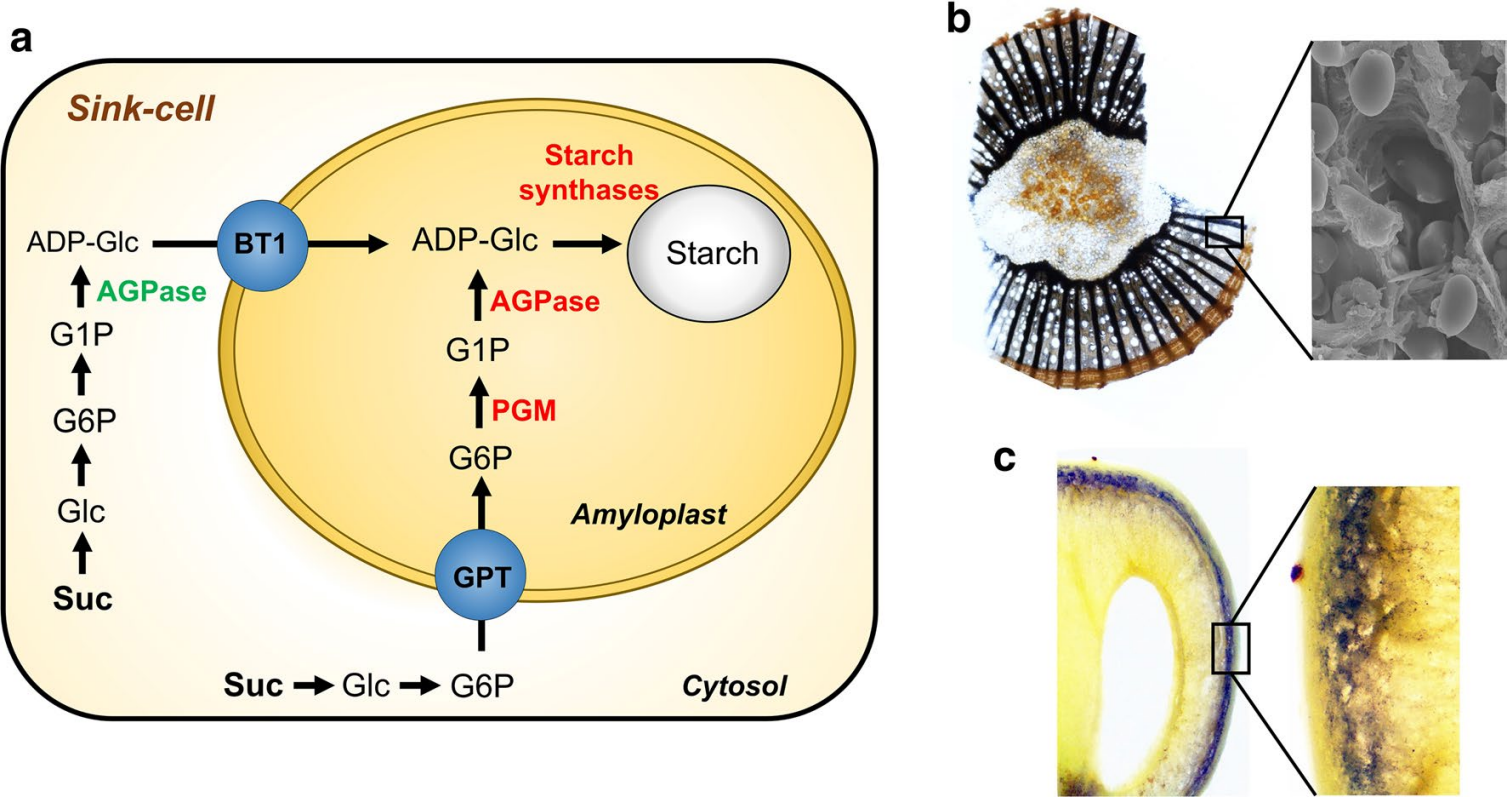
Starch metabolism and accumulation in heterotrophic tissues. **a** In heterotrophic tissues, G6P is translocated into the amyloplast by the GPT and converted into G1P by PGM. Following this reaction, G1P and ATP are combined into ADP-Glc, which is incorporated into the starch granule by starch synthases. In cereals, ADP-Glc is synthesized in the cytosol and is incorporated into the amyloplast by the BT1 protein. Starch accumulation in heterotrophic tissues of grapevine after iodine staining. **b** Starch accumulation in well-defined amyloplasts in xylem-ray cells, and in the outer layers of the mesocarp of green berries (**c**). *ADP* adenosine diphosphate, *ADP-Glc* adenosine diphosphate glucose, *AGPase* ADP-glucopyrophosphorylase, *ATP* adenosine triphosphate, *G1P* glucose-1-phosphate, *G6P* glucose-6-phosphate, *GPT* glucose-6-phosphate/phosphate translocator, *NTT* plastidic nucleotide transporter, *PGM* phosphoglucomutase, *Pi* inorganic phosphate, *Glc* glucose, *Suc* sucrose, *BT1* brittle 1

In contrast to chloroplasts, the synthesis and accumulation of reserve starch in heterotrophic plastids (amyloplast), found in roots, woody tissues, fruits, seeds, tubers, and pollen grains, relies on carbon obtained from long-distance sugar transport through the phloem (Figs. 1, 3; Lalonde et al. 2004; Lemoine et al. 2013). Although several biochemical steps of starch synthesis operating in leaves are conserved in heterotrophic tissues, some are particular to sink organs. For instance, in heterotrophic tissues, starch is synthesized in amyloplasts following the incorporation of glucose-6-phosphate (G6P) from the cytosol by a glucose-6-phosphate/phosphate translocator (GPT; Kammerer et al. 1998). This transmembrane protein was initially purified from the plastidial envelope membranes isolated from maize endosperm and, since then, several cDNAs have been isolated from different plant species (Kammerer et al. 1998). Functional studies in planta confirmed that GPT proteins are G6P transporters in Arabidopsis (Niewiadomski et al. 2005) and *Vitis vinifera* (Noronha et al. 2015). In grapevine, *VvGPT2* is more expressed in leaves and *VvGPT1* in heterotrophic tissues such as berries, canes and flowers. In cultured grape cells, *VvGPT1* expression was increased by ABA, light and galactinol, and *VvGPT2* by sucrose (Noronha et al. 2015). Some plants such as *Solanum tuberosum* may also incorporate glucose-1-phosphate (G1P) into the plastid to synthesize starch (Fettke et al. 2010).

ADP-glucopyrophosphorylase (AGPase) is present in both source and sink tissues and it is exclusively localized inside the plastid in all tissues except in the cereal endosperm, which despite possessing the plastic form most of its activity is cytosolic (Denyer et al. 1996; Burton et al. 2002). In cereals, adenosine diphosphate glucose (ADP-Glc) synthesized in the cytosol is translocated to the amyloplast by the inner envelope protein BT1 that, in maize, is encoded by the *Brittle1* gene (Fig. 3; Denyer et al. 1996; Beckles et al. 2001). In maize endosperm, this protein transports cytosolic ADP-Glc into the plastid in exchange for ADP (Sullivan and Kaneko 1995; Möhlmann et al. 1997; Kirchberger et al. 2007). Interestingly, in potato (Leroch et al. 2005) and Arabidopsis (Kirchberger et al. 2008) the BT protein is involved in the efflux of adenine nucleotides synthesized in the plastids. Also, GUS assays showed that in Arabidopsis AtBT1 is mainly expressed in developing anthers, in the central cylinder of young roots and root tips (Kirchberger et al. 2008).

## In cereal seeds α-amylase plays a pivotal role in starch mobilization

**Fig. 4:**
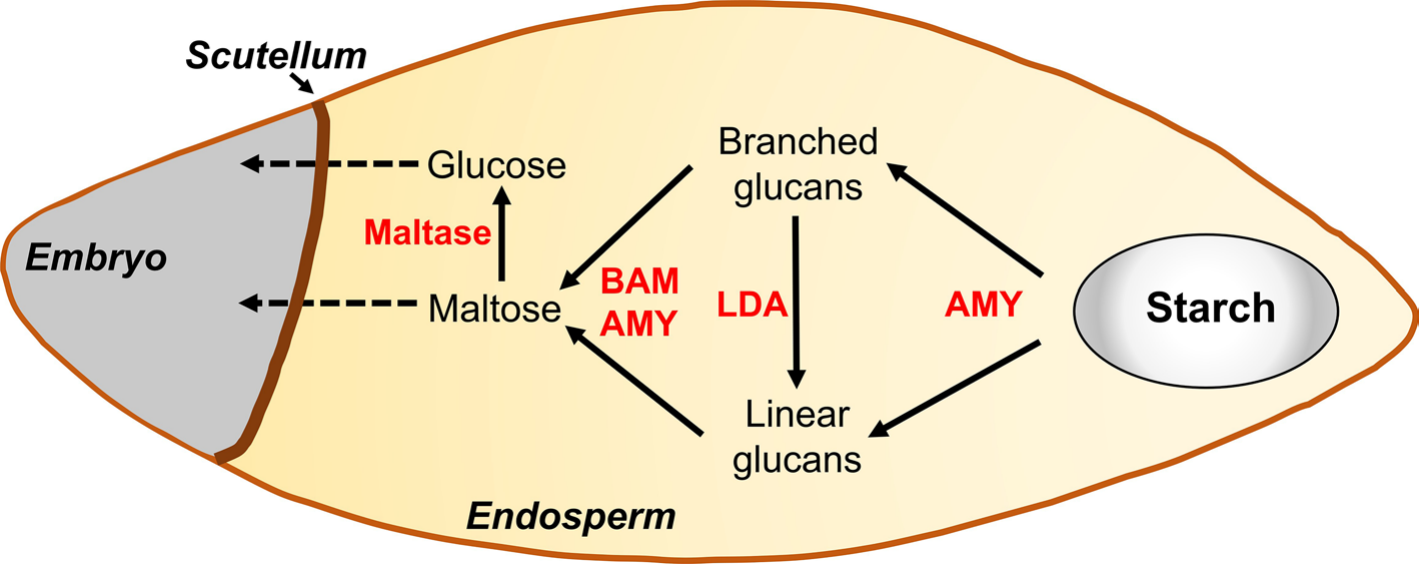
Starch mobilization in the cereal seed during germination. As the starchy endosperm is a dead tissue at the time of germination degrading enzymes are secreted by the embryo. The cooperative action of α- and β-amylases and limit dextrinase releases maltose units from the starch grain, which are hydrolyzed by maltases into glucose monomers and absorbed through the scutellum. *BAM* β-amylase, *AMY* α-amylase, *LDA* limit dextrinase Adapted from Andriotis et al. (2016)

Starch degradation in heterotrophic tissues has been most studied during the mobilization of reserves from the starchy endosperm during seed germination, although this process differs substantially from the one that occurs in autotrophic tissues (Zeeman et al. 2010). In mature cereal seeds, the starchy endosperm is essentially a dead tissue at the time of germination and is composed mainly of starch, cell wall polymers and storage proteins surrounded by the aleurone layer that is essential for starch mobilization. Starch is degraded primarily to glucose, following the action of AMYs, BAMs, LDA and maltases secreted by the scutellum and/or the aleurone layer, which is subsequently taken up by the embryo (Fig. 4; Andriotis et al. 2016b). Contrarily to what occurs in chloroplasts, in cereal seeds AMY plays a pivotal role in starch degradation (Zeeman et al. 2010). It initiates its degradation by releasing linear and branched glucose polymers from the granule, the latter being converted to linear glucans by LDA. The action of AMY and BAM on linear chains produces maltose and glucose. Finally, maltose is hydrolyzed by the action of maltases, which are specialized α-glucosidases that produce glucose for embryo growth. Inactive BAMs are deposited in the endosperm prior to the dehydration phase and are activated by proteases released by the aleurone (Radchuk et al. 2009). Interestingly, high BAM activity was likely selected during cereal domestication, because it is not necessary for starch degradation and germination of developing seeds, as shown by cereal mutants with low BAM activity (Daussant et al. 1981; Sun and Henson 1991; Kihara et al. 1999).

## Starch mobilization in woody tissues in winter for cold tolerance and in spring for bud burst

**Fig. 5:**
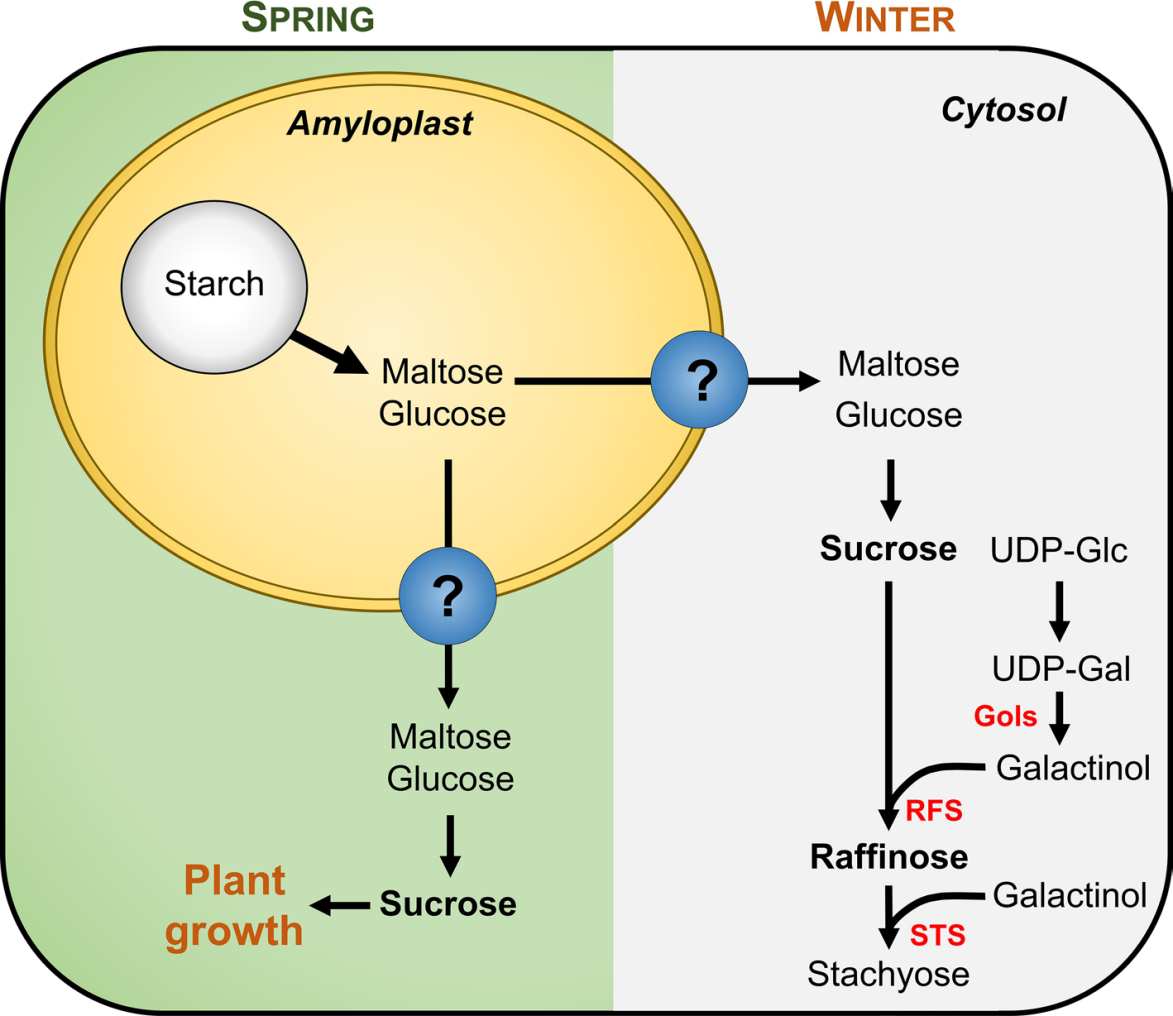
Starch mobilization in woody tissues during spring and in winter in response to cold. Starch reserves accumulated in woody tissues amyloplasts may be mobilized during spring to sustain plant growth and metabolism or during winter to allow the production of compatible solutes, mainly sucrose and raffinose, in response to cold temperatures. *UDP-Glc* uridine diphosphate-glucose, *UDP-Gal* uridine diphosphate-galactose, *Gols* Galactinol synthase, *RFS* raffinose synthase, *STS* stachyose synthase

Woody perennial trees must store carbon reserves to allow their survival during winter and for vegetative growth in the following spring (Fig. 5). Studies have shown two main periods of starch degradation in woody tissues—in the fall and spring before bud burst (Ashworth et al. 1993; Sauter and van Cleve 1994; von Fircks and Sennerby-Forsse 1998). The woody tissues function essentially as vegetative storage tissues in which starch accumulates seasonally in well-defined amyloplasts in the ray parenchyma cells (Sauter and van Cleve 1994). The localization of these starch-accumulating cells can be easily observed in a cross-section of a grapevine woody cane stained with Lugol’s iodine solution, as shown in Fig. 3.

During winter, the degradation of starch in woody tissues plays a crucial role in cold tolerance. The accumulation of sucrose and raffinose in response to cold stress has been described in several species, such as poplar (Sauter and van Cleve 1991, 1994), *Pinus strobus* L. (Hinesley et al. 1992), birch (Kasuga et al. 2007), and willow (Ögren 1999), which occurs concomitantly with the degradation of starch reserves (Sauter 1988; Witt and Sauter 1994a; Ögren 1999; Ashworth et al. 1993; Palonen et al. 2000). During spring, starch is mobilized to sustain plant growth, and in poplar woody tissues starch is completely hydrolyzed at the time of bud burst (Sauter and van Cleve 1994; Witt and Sauter 1994b).

Accordingly, an increase in the biochemical activity of starch-degrading enzymes has been reported in the period of starch–sugar conversion in woody tissues (Elle and Sauter 2000). The total amylolytic activity in poplar tissues is higher during the spring (Witt and Sauter 1994a) and autumn (Witt and Sauter 1994b), providing the carbon needed for sugar synthesis in the cytosol. Thus, sucrose or raffinose synthesis in the cytosol of woody tissues during cold adaptation and during spring depends of the transport of sugars from amyloplasts following starch breakdown. For instance, in *Ajuga reptans*, a species that accumulates large amounts of raffinose family oligosaccharides (RFOs) and translocates stachyose, most of the enzymes involved in raffinose synthesis are localized in the cytosol (Bachmann and Keller 1995), and similar results were obtained in spinach and Arabidopsis leaves exposed to cold treatment (Schneider and Keller 2009). Interestingly, the chestnut CsDSP4, a homolog of the Arabidopsis SEX4 phosphatase associated with starch degradation in leaves (Zeeman and Rees 1999; Niittylä et al. 2006), is induced in woody tissues during autumn starch catabolism to promote the accumulation of sugars (Berrocal-Lobo et al. 2011). Also, an increase in the activity of sucrose phosphate synthase, a cytosolic enzyme that synthesizes sucrose-6-phosphate from UDP-glucose and fructose-6-phosphate (Ito et al. 2011), has been reported in several plant species in response to cold treatments (Guy et al. 1992; Holaday et al. 1992; Hurry et al. 1995).

Thus, considering that the amyloplast maintains its integrity during starch degradation in the winter and spring (Sauter and van Cleve 1990; Sauter and van Cleve 1991), it is very likely that this process is different from the one that occurs in cereal seeds but similar to the one described in leaves at night. Therefore, during periods of starch mobilization, the sugars released by amylolytic enzymes inside the amyloplast must be translocated into the cytosol by the action of specific plastidic membrane proteins. It is tempting to speculate that the proteins involved in sugar export from the chloroplast, such as MEX and pGT, could also play a role in transporting maltose and glucose from the amyloplast. In fact, it has been reported that a MEX from apple is expressed not only in autotrophic tissues but also in immature leaves, roots and fruits (Reidel et al. 2008), and in grapevine, *VvMEX* is expressed in several heterotrophic tissues such as berries, flowers and canes (Noronha et al. unpublished). Also, a pGT from rice is highly expressed in the seeds during development (Toyota et al. 2006) and in olive tree it was found that a pGT is expressed and localized in tissues that do not accumulate starch (Butowt et al. 2003), suggesting broader physiological roles for these proteins than previously thought. Still, numerous physiological aspects of starch storage in woody plants and of its metabolization in spring and during winter are far from being understood (Geisler-Lee et al. 2006).

Also, the root system of woody plants is an important site of starch accumulation and its interplay with stem reserves is not yet fully understood, particularly under stress conditions that limit photosynthesis, where these reserves could be used to provide energy and carbon to the plant (Loescher et al. 1990; Zapata et al. 2004; Dovis et al. 2014; Thalmann and Santelia 2017). In grapevine potted plants, it was shown that the remobilization of root starch supplies sugars to the fruits in response to drought stress (Rossouw et al. 2017).

## Agronomical practices affect starch metabolism in woody plants

As shown above, in woody plants, each growing season merges with the previous and following ones through the carbohydrate reserves accumulated in woody tissues, making this topic of the outmost agronomical and scientific importance. In grapevine, delaying pruning after bud burst could deplete reserve carbohydrates and the reiteration of this practice may cause carry-over effects on vine vigor and yield (Moran et al. 2017; Petrie et al. 2017). Similarly, trunk girdling, a practice that limits phloem transport of photoassimilates to the roots while maintaining xylem water flow, affects the replenishment and mobilization of carbohydrate reserves (Roper and Williams 1989; Mei et al. 2015). Also, defoliation reduces the accumulation of carbohydrate reserves in woody organs, with early ones having the largest impact (Bennett et al. 2005; Smith and Holzapfel 2009; Zufferey et al. 2012). For example, in grapevine, source–sink ratio that may be decreased by partial defoliation or increased by cluster thinning, are normally associated with several modifications of vineyard efficiency and grape and wine quality. With the aim to decipher the underlying mechanisms, we have recently evaluated how the common agricultural practice of leaf removal may modulate at the molecular level the storage of starch in grapevine woody canes. Our results (Silva et al. 2017) showed that severe leaf removal promotes a consistent decrease in starch, sucrose and phenolics that accumulate in 1-year-old canes. At the molecular level, alterations of source–sink ratios resulted in a transcriptional adjustment of genes involved in starch metabolism, including and upregulation of *VvGPT1* and *VvNTT* (plastidic ATP/ADP translocator) for higher cluster/leaf ratios (Silva et al. 2017). Recently, it was shown that under reduced water supply during berry ripening, starch remobilization from roots is concurrent with rapid berry sugar accumulation and intensified by defoliation (Rossouw et al. 2017). Manipulation of source–sink relationship by fruit thinning can contribute to maintain high levels of carbohydrate reserve in woody organs. In grapevine, fruit thinning at the onset of ripening increases total non-structural carbohydrate concentration in the roots in subsequent seasons (Smith and Holzapfel 2009). Furthermore, fertilization may have an impact in the accumulation of starch reserves, as mineral nitrogen (N) supply was shown to decrease non-structural carbohydrates in apple trees (Cheng and Fuchigami 2002). It has been reported that starch accumulated higher in woody tissues of apple trees growing in water culture without N supply and amylase, sucrose-6P synthase and sucrose synthase activities of wood and bark tissues were suppressed by N deprivation (Yoshioka et al. 1989).

## Conclusion

Food scarcity due to an increase in the global population is a problem that is a challenge for scientists and policy makers, thus, unraveling the mechanisms of starch synthesis in both auto- and heterotrophic tissues is of the utmost scientific and societal importance. As shown in this review, several enzymes and plastidic proteins, such as MEX and pGT, modulate carbon allocation in plant tissues, but their role in starch mobilization in sink organs, such as woody tissues, deserves further investigation. Several experimental constrains, including the lack of model plants amenable for transformation and the absence of mutants, limit the study of these processes in woody tissues. Nevertheless, the advances that have been made in the understanding of starch metabolism in Arabidopsis and maize, together with the increasing availability of next-generation sequencing tools, will allow researchers to obtain a better picture of the molecular processes that occur in woody tissues.

### *Author contribution statement*

HG and HN conceived the idea of the review and prepared the first draft. All authors added new information to the manuscript, read and approved the final version.
