# Correction to: A molecular perspective on starch metabolism in woody tissues

**DOI:** 10.1007/s00425-018-2963-1

**Published:** 2018-08-02

**Authors:** Henrique Noronha, Angélica Silva, Zhanwu Dai, Philippe Gallusci, Adamo D. Rombolà, Serge Delrot, Hernâni Gerós

**Affiliations:** 1 Centre of Molecular and Environmental Biology (CBMA), University of Minho, Braga, Portugal; 2 UMR EGFV, Bordeaux Science Agro, INRA, Université de Bordeaux, Villenave D’Ornon, France; 3 Department of Agricultural Sciences, Alma Mater Studiorum, University of Bologna, Bologna, Italy; 4 Centro de Investigação e de Tecnologias Agro-ambientais e Biológicas (CITAB), Vila Real, Portugal; 5 Centre of Biological Engineering (CEB), University of Minho, Braga, Portugal

## Correction to: Planta 10.1007/s00425-018-2954-2

The article “A molecular perspective on starch metabolism in woody tissues”, written by Henrique Noronha, Angélica Silva, Zhanwu Dai, Philippe Gallusci, Adamo D. Rombolà, Serge Delrot, and Hernâni Gerós, was originally published electronically on the publisher’s internet portal (currently SpringerLink) on 18 July 2018 without open access. With the author(s)’ decision to opt for Open Choice the copyright of the article changed on 1 August 2018 to © The Author(s) 2018 and the article is forthwith distributed under the terms of the Creative Commons Attribution 4.0 International License (http://creativecommons.org/licenses/by/4.0/), which permits use, duplication, adaptation, distribution and reproduction in any medium or format, as long as you give appropriate credit to the original author(s) and the source, provide a link to the Creative Commons license and indicate if changes were made.

The original article was corrected.

